# Case Report: Can Inhaled Adenosine Attenuate COVID-19?

**DOI:** 10.3389/fphar.2021.676577

**Published:** 2021-08-09

**Authors:** Bruce D. Spiess, Michael Sitkovsky, Pierpaolo Correale, Nikolaus Gravenstein, Cynthia Garvan, Timothy E. Morey, Brenda G. Fahy, Leslie Hendeles, Thomas J. Pliura, Thomas D. Martin, Velyn Wu, Corey Astrom, Danielle S. Nelson

**Affiliations:** ^1^ Department of Anesthesiology, University of Florida College of Medicine, Gainesville, FL, United States; ^2^ New England Inflammation and Tissue Protection Institute - Northeastern University, Boston, MA, United States; ^3^ Medical Oncology Unit, Covid19 Scientific Task Force, Grand Metropolitan Hospital, Reggio Calabria, Italy; ^4^ Department of Anesthesiology, University of Florida School of Medicine, Gainesville, FL, United States; ^5^ College of Pharmacy, University of Florida, Gainesville, FL, United States; ^6^ Private Practice Emergency Department, Champaign, IL, United States; ^7^ Department of Surgery (Cardiac Surgery), University of Florida School of Medicine, Gainesville, FL, United States; ^8^ Department of Community Health and Family Medicine, University of Florida, College of Medicine, Gainesville, FL, United States

**Keywords:** COVID-19, acute respiratory distress syndrome, ARDS, nebulizer, adenosine, case report

## Abstract

This case report demonstrates a small repetition of the case series carried out in Italy wherein inhaled adenosine was administered to patients experiencing severe and worsening coronavirus disease-2019 (COVID-19). The two cases are important not only because they were the first of their type in the United States, but also because both patients were DNR/DNI and were therefore expected to die. Study repetition is vitally important in medicine. New work in pharmacology hypothesizes that adenosine-regulator proteins may play a role in the pathogenesis of COVID-19 infection. Furthermore, adenosine, by interacting with cell receptor sites, has pluripotent effects upon inflammatory cells, is anti-inflammatory, and is important in tissue hypoxia signaling. Inhaled adenosine is potentially safe; thousands have received it for asthmatic challenge testing. The effects of adenosine in these two cases were rapid, positive, and fit the pharmacologic hypotheses (as seen in prior work in this journal) and support its role as a therapeutic nucleoside.

## Introduction

We discuss two cases that presented with profound respiratory distress a number of days after contracting COVID-19 pneumonia. Both patients had polymerase chain reaction (PCR)-confirmed COVID-19 and both patients had vital signs with tachycardia and respiratory rates >30 breaths per minute. Furthermore, each person presented with low peripheral pulse oximetry levels, 85 and 64, respectively. Patient one was on high-flow oxygen and Patient two was on oxygen when these presenting vital signs were found. Arterial blood gases obtained in Patient one showed a PaO_2_ of 52 mmHg and in Patient two (after establishment of oxygen therapy), a PaO_2_ of 48 mmHg. These two cases are unique in that both patients were likely to die in a short period of time but both were offered off-label inhaled adenosine as a therapeutic option. Both recovered and these cases are reported herein because of the rapid response seen, as well as the fact that these first-in-the-United States treatments demonstrate a repetition of groundbreaking work from Italy.

**FIGURE 1:**
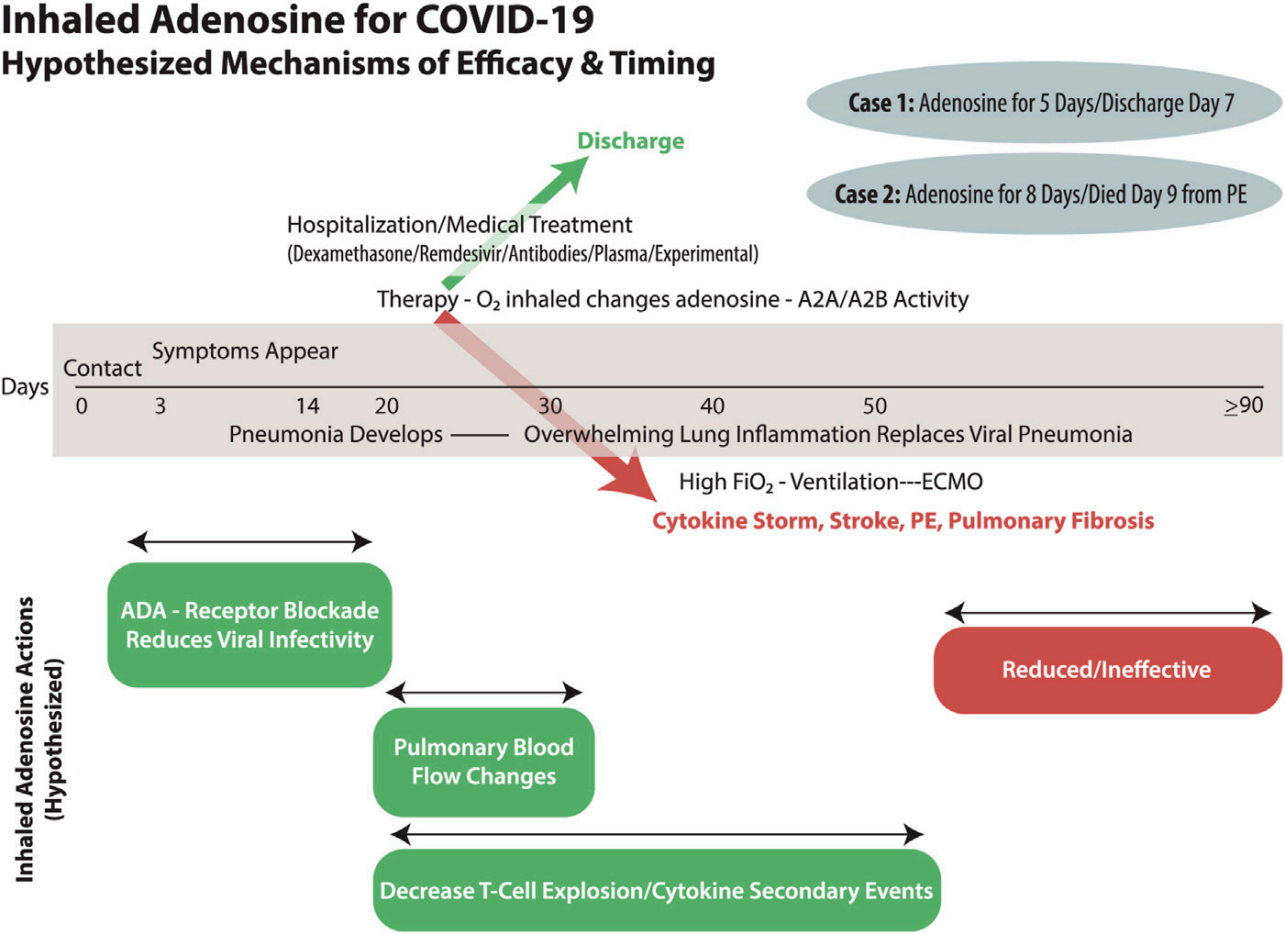
COVID-19 has an individualized and varied timeline from exposure through nasopharyngeal infection, pulmonary spread, pneumonia, and then secondary ARDS. The involvement of oxygen therapy needs to be explored as both a therapeutic and causative player in ARDS as well as the explosive inflammatory cytokine storm of events. The timely use of inhaled adenosine may have efficacy in treatment of COVID-19 infection and secondary complications depending upon the multiple mechanisms by which it could act. Our two patients had a rapid response that almost certainly must have been due to pulmonary blood flow issues, but they recovered from severe COVID-19 ARDS with consecutive days of inhaled therapy. Eventually, and late in the ARDS lung fibrosis continuum, it must be that inhaled adenosine will have reduced effectiveness.

Acute respiratory distress syndrome (ARDS) occurs in 3–5% of patients with COVID-19 who have undergone varying degrees of supplemental oxygen therapy, sometimes leading to mechanical ventilation (Pascarella et al., 2020; Yang et al., 2020). Prior work published in this journal (Geiger et al., 2020) proposed three hypotheses regarding coronaviruses and adenosine. First proposed was that COVID-19 might gain access to pneumocytes through attachment to adenosine kinase (ADK) and adenosine deaminase (ADA) after adhering to viral spike proteins to the angiotensin-converting enzyme (ACE) on cell surfaces. The adenosine-processing transmembrane proteins (ADK and ADA) are coupled with dipeptidyl peptidase 4 (Geiger et al., 2020). Middle Eastern Respiratory Syndrome-CoV (MERS-COV) has been shown to gain cell entrance through this mechanism of adenosine binding site pore intrusion (Raj et al., 2014; Geiger et al., 2020). COVID-19 and MERS are similar (Raj et al., 2014; Geiger et al., 2020). Extracellular adenosine competes with MERS-CoV for ADK/ADA enzyme binding and transmembrane flow. If such coronaviruses viruses gain intracellular access, they replicate (an adenosine triphosphate/adenosine-consuming reaction) (Geiger et al., 2020). Intracellular replication kills the host cell by degrading energy stores, consuming adenosine, and degrading proteins. Widespread inflammation of pneumocytes leads to an influx of tissue macrophages and killer T-cells, with tremendous secondary inflammation in efforts to ingest and quell the virus, as well as efforts to mop up dead pneumocytes (Geiger et al., 2020). Adenosine is known to down-regulate killer T-cell activity and decrease platelet activation, as well as produce vasodilation and change pulmonary blood flow. Our case report and the Italian series demonstrate clinical efficacy in patients who were in extremis, likely to die, and who were already receiving the best therapy with contemporary regimens of therapy for COVID-19 (Correale et al., 2020). (Figure 1) These cases are compelling and create demand for research, cases series, and randomized controlled trials (timing, dosing, etc.) into efficacy and mechanism (which these cases were unable to investigate).

Both patients (or family) in our case series provided written consent for publication, per University of Florida Institutional Review Board protocol.

## Case 1

An 89-year-old woman with a history of coronary artery disease, myocardial infarction, polymyalgia rheumatica, temporal arteritis, bronchiectasis, and interstitial lung disease presented with dyspnea and myalgias after 10 days of illness. A computed tomography scan of the lungs revealed extensive reticular infiltrates in the lower lobes intermixed with ground-glass opacities. She was diagnosed with COVID-19 by nasal swab-obtained samples for PCR.

She was admitted and given azithromycin and ceftriaxone for presumed bacterial pneumonia. Her SpO_2_ on admission was 94% on 2 L nasal cannula (NC) O_2_ and she was started on remdesivir and dexamethasone (Day 1). On hospital Day 3, increased work of breathing, hypoxia, and worsening non-productive cough occurred, leading to further treatment for superimposed bacterial pneumonia. Her SpO_2_ was 87% despite 3.5–6 L/min O_2_ and a PaO_2_ of 59 mmHg over Days 3 to 4. Oxygen support was advanced to a non-rebreathing mask (Day 4; FiO_2_ 50%) with a resultant PaO_2_ of 78 mmHg. The patient and family requested a Do Not Resuscitate/Intubate status on Day 4. High-flow NC O_2_ (60 L/min at 70% FiO_2_) was accompanied by a respiratory rate >30 breaths per minute on Day 7 with an arterial blood gas PaO_2_ of 59 mmHg. She could barely speak. On Day 8, the patient consented to off-label use of inhaled adenosine at 9 mg in 3 mL of normal saline administered by a vibrating mesh nebulizer (Aerogen, Galway, Ireland) linked to high-flow 21% O_2_. Upon administration of adenosine (5 min), her SpO_2_ improved to 97% with a concomitant decrease in FiO_2,_ from 75 to 21%. She reported feeling as though her lungs were “opening up,” and her respiratory rate went from 30 to 40/min to 18 to 22/min. She was hemodynamically stable without bronchoconstriction or flushing, and she received another inhaled adenosine treatment 12 h later and then once daily for the next 4 days without apparent side effects. By Day 2 of adenosine treatment, she was eating and sleeping, and was ambulating by Day 4. The patient was discharged home 7 days after adenosine commenced. She is now off oxygen and with her family and recovered.

## Case 2

A 64-year-old man in rural Illinois with chronic myelocytic leukemia and secondary anemia (Hgb 6–7 g/dL) developed COVID-19. His family, members of an Apostolic Christian faith, limit use of traditional medical care, but had a relationship with a neighbor who was a local emergency room doctor. The patient, ill for 1 week, was in extremis when the family called the doctor, who made a home visit and brought a pulse oximeter and rapid COVID test (positive and later confirmed by PCR). When the doctor arrived at the farmhouse, the patient was breathing at 40 breaths per minute and was cyanotic (SpO_2_ 67% on room air). He declined hospitalization. The doctor had knowledge of the Italian/UF experiences. He conferred with the UF team, deciding that extreme situations deserve extreme measures. The patient was treated at the farm with oxygen by close-fitting mask, adenosine (9 mg, nebulized), dexamethasone, and antibiotics. Immediately upon treatment, the patient’s SpO_2_ improved to 87% and his breath rate to 20 per minute. A second dose of adenosine was given at 12 h, and continued for 7 days with twice daily treatments. On Day 4, the patient was eating but was anemic (5.8 g/dL), weak, and was convinced to receive red blood cells and a chest X-ray as an outpatient in an emergency department (bilateral consolidating pneumonia symptomatic of ARDS). On Day 8, he was on 2–5 L NC oxygen with an SpO_2_ of 92–95%. On Day 9, the patient walked to his barn to check on farming operations. He felt acutely dyspneic with pleuritic chest pain, was carried to the farmhouse, refused hospitalization, became tachycardic (115–125 beats per minute) with an SPO_2_ of 70%, and was gasping—an acute change in minutes. He died quickly with a presumptive diagnosis of pulmonary embolism secondary to COVID-19. The family refused an autopsy.

## Discussion

Our two patients responded to adenosine therapy. We were unable to perform hypothesis testing or adenosine level measurements because these two cases were done as an off-label clinical effort to save human life and frankly they were not consented for such testing. That these two cases (albeit a very small number) in a different healthcare system provide an apparent parallel effect to that described in Italy (Correale et al., 2020) is important and notable in that repetition of positive results in medicine is vital. Together, our cases, plus the published Italian work (Correale et al., 2020), press the need for hypothesis-based research, toxicologic testing, and prospective randomized trials. This case report cannot satisfy the desire for more mechanistic and pharmacologic information at this time. The immediate improvement we experienced was likely due to pulmonary blood flow changes and not a reduction in inflammation. But the ultimate recovery from ARDs, we believe, has its basis in down-regulation of T-cells, as previously described (Ohta and Sitkovsky, 2001; Sitkovsky et al., 2004; Bours et al., 2006; Aggarwal et al., 2013; Nowak-Machen et al., 2013; Hatfield et al., 2014; Sharma et al., 2016). The death of the patient in Case 2, presumed a pulmonary embolism, was clearly not an ARDS demise. Yet it must be stated that we cannot rule out an interaction with inhaled adenosine with his COVID-19 disease and/or some unforeseen toxic effect. Clinical trials research needs to be done immediately. Treatment with inhaled adenosine for patients with a PaO_2_/FiO_2_ ratio of <300 and at the point of imminent mechanical ventilation could accelerate recovery and save lives. The strengths of such an approach with adenosine are many. The drug is widely available, off patent, and can be easily administered. It appears safe, yet toxicology of repeated daily inhalation has not been rigorously conducted. If the inhaled adenosine is anti-viral early on, then it could open up new and perhaps far-reaching anti-viral technology.

Adenosine, a pluripotent RNA nucleoside, is exported from cells in response to hypoxia-inducible factor-1-α–sensed local tissue hypoxia (Ohta and Sitkovsky, 2001; Sitkovsky et al., 2004; Bours et al., 2006; Aggarwal et al., 2013; Nowak-Machen et al., 2013; Hatfield et al., 2014; Sharma et al., 2016; Widysanto et al., 2020). Adenosine, a building block of energy metabolism, is vital to normal physiology, it is anti-inflammatory, and it modulates vascular tone as well as localized tissue blood flow (Ohta and Sitkovsky, 2001; Sitkovsky et al., 2004; Bours et al., 2006; Aggarwal et al., 2013; Nowak-Machen et al., 2013; Hatfield et al., 2014; Sharma et al., 2016; Widysanto et al., 2020). It is vital to energy production and storage through phosphorylated di- and triphosphate molecules in the Krebs cycle. Cells use this energy, but particularly erythrocytes carry a great deal of adenosine and release it at sites of low flow or upon cellular shear and in so doing, autoregulate localized blood flow. Various proteins and nucleoside transporters are important in the constant flux in and out of cells as well as the maintenance, metabolism, and equilibrium of extracellular and intracellular levels of adenosine (Ohta and Sitkovsky, 2001; Sitkovsky et al., 2004; Bours et al., 2006; Aggarwal et al., 2013; Nowak-Machen et al., 2013; Hatfield et al., 2014; Raj et al., 2014; Sharma et al., 2016; Correale et al., 2020; Geiger et al., 2020; Widysanto et al., 2020).

Of interest and fitting with the hypothesis (Geiger et al., 2020) that extracellular adenosine interactions at various receptor sites are vital in the role of infection and lethality of COVID-19 and MERS-CoV (Geiger et al., 2020) is new work in cystic fibrosis (CF) (Abraham et al., 2021). Adenosine levels have been tested in patients with CF (Abraham et al., 2021) showing that they have abnormally high extracellular adenosine levels due to cellular leakage (10 to 100-fold normal) (Abraham et al., 2021). A patient with CF’s propensity for pneumonia and lung supra-infection might make him/her highly susceptible to COVID-19 with an expected very high mortality rate in the population. However, the opposite is true. In multiple countries, no deaths from COVID-19 have occurred in patients with CF. It has been postulated that patients with CF have resistance to COVID-19 because they leak adenosine from inside their cells due to a dysfunction of transmembrane peptidases (where MERS-CoV binds) (Abraham et al., 2021). Furthermore, it is hypothesized that the natural and high concentrations of adenosine in patients with CF lead to A2A and A2B receptors recognizing more anti-inflammatory signals than those seen in patients without CF. Interestingly, the elderly and those with many underlying chronic disease states have low extracellular adenosine levels and an inability to increase adenosine/ATP export from their cells (Abraham et al., 2021). The CF data, the Italian findings, and our patients fit with the mechanisms and therapeutic options put forward herein and in the prior *Frontiers in Pharmacology* paper (Geiger et al., 2020).

Local adenosine flux or reduction in the lung prompts vasoconstriction and increases pulmonary shunt. Increased FiO_2_ administered to treat hypoxia interferes with the natural hypoxia-inducible factor-1-α–adenosine axis (Ohta and Sitkovsky, 2001; Sitkovsky et al., 2004; Bours et al., 2006; Aggarwal et al., 2013; Nowak-Machen et al., 2013; Hatfield et al., 2014; Sharma et al., 2016; Dhont et al., 2020; González-Duaarte and Nordcliffe-Kaufmann, 2020; Widysanto et al., 2020). Exogenous administration of adenosine might well allow for restoration of pulmonary adenosine signaling through A2A receptors while relieving systemic hypoxia. “Happy hypoxia” in COVID-19 is noted in some patients who do better with delayed intubation or lower FiO_2_ (Couzin-Frankel, 2020; Dhont et al., 2020; González-Duaarte and Nordcliffe-Kaufmann, 2020; Widysanto et al., 2020). This phenomenon may be explained by the adenosine axis and A2A receptors—that by having permissive relative hypoxia, patients suffering from COVID-19 ARDS have actually increased their endogenous pulmonary adenosine.

Adenosine fits the needs of COVID-19-caused ARDS (Ohta and Sitkovsky, 2001; Sitkovsky et al., 2004; Bours et al., 2006; Aggarwal et al., 2013; Nowak-Machen et al., 2013; Hatfield et al., 2014; Sharma et al., 2016; Widysanto et al., 2020). Extracellular adenosine has powerful cell signaling, anti-inflammatory, anti-platelet, and anti-T cell effects. Normal plasma concentrations range from 30 to 300 nM, but with hypoxia, they elevate to 600–1,200 nM. Adenosine affects cellular G-protein-linked receptors (A2A and A2B), interfering with toll-like receptor 4-mediated responses that mitigate lymphocyte cytokine responses (Ohta and Sitkovsky, 2001; Sitkovsky et al., 2004; Bours et al., 2006; Aggarwal et al., 2013; Nowak-Machen et al., 2013; Hatfield et al., 2014; Sharma et al., 2016; Widysanto et al., 2020).

Adenosine, a potent platelet inhibitor (Sharma et al., 2016), could potentially affect microthrombosis and diffuse intravascular coagulation. It modulates macrophages, enhancing cellular repair mechanisms and vascular endothelial growth factor (Haskó and Pacher, 2012; Fuentes et al., 2014; Boncler et al., 2019; Widysanto et al., 2020). Reduction of viral loads (as seen in the Italian series) with adenosine has also been reported in West Nile and flaviviruses in conjunction with adenosine usage (Eyer et al., 2017; Tchnesokov et al., 2019). Remdesivir is 1’-cyano-substituted adenosine and shows broad-spectrum anti-viral activity (Tchnesokov et al., 2019).

Inhaled adenosine acts differently than intravenous adenosine (Fowler et al., 2000; Fardon et al., 2004; Hendeles et al., 2015a; Hendeles et al., 2015b; Daley-Yates et al., 2020) and does not create a peak blood effect akin to an intravenous bolus. No hemodynamic effects have been described with this technique. Inhaled adenosine has been widely used “off label” as an inhaled diagnostic test of asthma and as a bioassay of inhaled corticosteroid therapy (Fowler et al., 2000; Fardon et al., 2004; Hendeles et al., 2015a; Hendeles et al., 2015b; Daley-Yates et al., 2020). Dosages 10-fold or greater than what we administered have been well tolerated in bronchoprovocation studies (Hendeles et al., 2015a). Inhaled adenosine is so safe that it has been used in thousands of patients—even in pediatric outpatient offices.

Thus far, adenosine appears safe, yet full toxicology of repeated daily inhalation has not been worked out. If inhaled adenosine is anti-viral early on, then it could open up new and perhaps far-reaching anti-viral technology. Yet, limitations may exist. At this point, these studies amount to small case series with exciting hypothesized mechanisms. A great deal of effort with randomized controlled trials is required to make sure the observations are validated, and considerable work is yet to be done to confirm the mechanistic hypotheses put forth in this journal and by other work from the NIH. The use of inhaled adenosine is “off label” and not approved by either the European Regulatory Commission or the United States FDA at this time. If this technique is safe and even partially effective before hospitalization, it represents a monumental advance in stopping the spiraling deaths due to COVID-19 ARDS. Clinical practice guidelines could rapidly result from “off label” successes in conjunction with early randomized trials. Toxicology efforts for repetitive inhaled adenosine are under appeal to funding agencies. An Investigational Drug Application has been applied for at the FDA, with toxicology being needed. Future work for other ARDS events beyond COVID-19 are compelled to be performed with this technology if continued encouraging results accrue. The multiple mechanistic hypotheses (Geiger et al., 2020) of inhaled adenosine efficacy compel rigorous exploration.

Table 1 provides a snapshot of treatment for both patients.

**TABLE 1:** Characteristics of two patients treated with inhaled adenosine. Both were elderly/older and both had pre-existing medical conditions.

Variable	Case 1	Case 2
Age	89	64
Sex	F	M
Past medical history	Coronary artery disease, myocardial infarction, polymyalgia rheumatica, temporal arteritis, primary interstitial pulmonary fibrosis	Chronic myelogenous leukemia treated with homeopathic medicine
Allergies	None	None
COVID treatment	Remdesivir, dexamethasone, azithromycin	Dexamethasone and azithromycin
Oxygen therapy prior to inhaled adenosine	70% O_2_ high-flow nasal cannula at 70 L	None, room air
Respiratory rate prior to adenosine	25–35 BPM	35–45 BPM
Treatment with adenosine (9 mg in 3 ml normal saline at each treatment)	BID on Day 1, Once per day for 4 more days	BID for 7 days

### Patient Perspective

Both patients and their families expected death within hours or a day. They were approached by physicians and the therapy was discussed frankly with the perspective that this had been tried in Italy with some success. One family was quite sophisticated in their medical background and the other less so but a basic summary of the science behind why an adenosine inhalant might work was presented. Neither patient could concentrate or comprehend the scientific discussion but both were given as much information as they could attend and take in. Respiratory distress and impending death for both patients may well have played a psychological part in their immediate and willing consent to undergoing the inhalant treatment. Neither patient was able to form sentences, speak, or converse with the physicians prior to the first treatment. Within 5 min of inhaled adenosine, Patient one expressed (later): “I could feel my lungs opening up and I could breathe again.” Indeed, by the end of the first adenosine treatment, she was able to speak in short but full sentences. Similarly, Patient two noted that he could speak and converse with his physician after the first treatment. He could now sit up in a chair and “catch” his breath. Each patient steadily improved each day of treatment. By Day 4, Patient one was eating and by Day 5, Patient two was ambulating, eating, and interacting with his family. Notably, Patient two underwent treatment at his home, interacting with his family the entire time of treatment. Patient one went home on Day 7 and has been communicative with her team, expressing thanks for being alive and for the adenosine therapy. Patient two died on Day 9 after therapy, but he died doing something he wished: farming and interacting with his sons in his barn. His family expressed deep gratitude to the treating physician for not only trying something off label, but they also felt their father’s dramatic recovery was a blessed event. Both patients and families noted that the adenosine inhalation was easy, non-painful, and created both immediate and slowly improving conditions.

## Data Availability

The original contributions presented in the study are included in the article/Supplementary Material, further inquiries can be directed to the corresponding author.
